# Supplementary material for: Inhibition of PIKfyve kinase prevents infection by Zaire ebolavirus and SARS-CoV-2
Source: Proc Natl Acad Sci U S A. 2020 Aug 6;117(34):20803–13. doi: 10.1073/pnas.2007837117 (PMC7456157; doi:10.1073/pnas.2007837117)
Supplement: Supplementary File [file pnas.2007837117.sapp.pdf]

Video 1. Apilimod doesn't inhibit VSV-MeGFP entry. Maximal Z-projection from four optical sections separated 0.25 μm apart of SVG-A cells gene-edited to express TagRFP-Rab5c imaged by spinning disc confocal microscopy every 3 seconds for 3 min. Cells were infected with VSV-MeGFP (MOI = 4) in the presence of CHX with or without 5 μM Apilimod and imaged ~ 3-4 h post-infection.

Video 2. Apilimod inhibits VSV-MeGFP-ZEBOV entry. Maximal Z-projection from four optical sections separated 0.25 μM apart of SVG-A cells gene-edited to express TagRFP-Rab5c imaged by spinning disc confocal microscopy every 3 seconds for 3 min. Cells were infected with VSV-MeGFP-ZEBOV (MOI = 3) in the presence of CHX with or without 5 μM Apilimod and imaged ~ 6-7 h post infection.

Video 3. Apilimod inhibits VSV-MeGFP-ZEBOV entry. Maximal Z-projection from four optical sections separated 0.25 μM apart of SVG-A cells gene-edited to express NPC1-Halo imaged by spinning disc confocal microscopy every 3 seconds for 3 min. Cells were infected with VSV-MeGFP-ZEBOV (MOI = 3) with or without 5 μM Apilimod and imaged ~ 5 h post infection.

www.pnas.org/cgi/doi/10.1073/pnas.2007837117
